# Real-Time Expanded Field-of-View for Minimally Invasive Surgery Using Multi-Camera Visual Simultaneous Localization and Mapping

**DOI:** 10.3390/s21062106

**Published:** 2021-03-17

**Authors:** Ahmed Afifi, Chisato Takada, Yuichiro Yoshimura, Toshiya Nakaguchi

**Affiliations:** 1 Department of Computer Science, College of Computer Science and Information Technology, King Faisal University, P.O. Box 400, Al-Ahsa 31982, Saudi Arabia; 2 Faculty of Computers and Information, Menoufia University, Menoufia 32511, Egypt; 3 Graduate School of Science and Engineering, Chiba University, Chiba 263-8522, Japan; takadachisato@chiba-u.jp; 4 Center for Frontier Medical Engineering, Chiba University, Chiba 263-8522, Japan; yysmr@chiba-u.jp

**Keywords:** field-of-view expansion, image mosaicking, multi-camera vSLAM, trocar with camera (CARET), computer-aided surgery

## Abstract

Minimally invasive surgery is widely used because of its tremendous benefits to the patient. However, there are some challenges that surgeons face in this type of surgery, the most important of which is the narrow field of view. Therefore, we propose an approach to expand the field of view for minimally invasive surgery to enhance surgeons’ experience. It combines multiple views in real-time to produce a dynamic expanded view. The proposed approach extends the monocular Oriented features from an accelerated segment test and Rotated Binary robust independent elementary features—Simultaneous Localization And Mapping (ORB-SLAM) to work with a multi-camera setup. The ORB-SLAM’s three parallel threads, namely tracking, mapping and loop closing, are performed for each camera and new threads are added to calculate the relative cameras’ pose and to construct the expanded view. A new algorithm for estimating the optimal inter-camera correspondence matrix from a set of corresponding 3D map points is presented. This optimal transformation is then used to produce the final view. The proposed approach was evaluated using both human models and in vivo data. The evaluation results of the proposed correspondence matrix estimation algorithm prove its ability to reduce the error and to produce an accurate transformation. The results also show that when other approaches fail, the proposed approach can produce an expanded view. In this work, a real-time dynamic field-of-view expansion approach that can work in all situations regardless of images’ overlap is proposed. It outperforms the previous approaches and can also work at 21 fps.

## 1. Introduction

In most cases, minimally invasive surgery is preferred over traditional open surgery because it reduces patient burdens. Specifically, laparoscopic surgery [1] has attracted a great deal of attention. In this surgery, the laparoscope and the forceps are inserted through 5 or 12 mm holes made around the surgery site. Therefore, the surgical wound and postoperative pain are small compared to conventional open surgery, and early discharge and early return to society are possible. Despite these advantages, due to the narrow angle of view and the dependence on a single laparoscope image, the burden on surgeons increases. Additionally, due to concerns about medical injuries in laparoscopic surgery that have occurred in recent years, there is a strong demand for improving the safety of laparoscopic surgery. As one of the solutions, it is important to provide an expanded surgical view while maintaining minimal invasiveness.

In the literature, there are many attempts to extend the surgical field of view using image mosaicking and mapping techniques. In [2], image mosaicking was used to construct a panoramic image of the bladder from a set of images obtained from an endoscope. The authors used mutual information to initially align all images and afterwards all transformation was adjusted to enhance the visibility of the final panoramic image. This method is useful for providing a static view; however, it cannot provide a real expanded view in real time. Additionally, images’ overlap is essential for image registration. A similar method for enhancing the surgical view in Natural Orifice Transluminal Endoscopic Surgery (NOTES) was also proposed in [3]. Vemuri et al. [4] extended the conventional mosaicking approach to construct a panoramic image from a sequence of laparoscopic images. In their approach, feature extraction and matching between consequent images are utilized to estimate planar homography transformations. These transformations are recursively combined to map the current frame to the reference frame. Bundle adjustment is then used to reduce the accumulated error. In their method, a static panoramic image can be constructed, and it suffers from the same limitation of the previous methods. Image feature tracking was also utilized in [5] to estimate image-to-image transformation and to construct an extended view by stitching together several video frames. This method can work at an average rate of 7.5 fps. Several similar stitching methods can be found in this review article [6].

The authors of [7,8] used simultaneous localization and mapping to provide an expanded surgical view. They built a 3D model of the surgical site based on the laparoscope navigation. In their method, this 3D model is utilized to augment the current laparoscope view with parts from outside the current view. A virtual camera located in the same location as the laparoscope is used to render the extended view. These methods advance one step further as they integrate the current laparoscope view with the static view obtained from a 3D model. However, these methods depend on a single laparoscope image and cannot provide multiple views at the same time. Another promising attempt to expand the surgical field of view was presented in [9]. In their method, two small cameras are attached to the main laparoscope to capture a wider surgical view. Images captured by the laparoscope and cameras may be combined to produce an expanded view; however no results were provided. Moreover, because the cameras are attached to the laparoscope, they move together and can capture the areas around it only. Mahmoud et al. [10] proposed a dense 3D reconstruction approach to enhance laparoscopic surgery. Although the results of this approach are promising, it is based on a single view and 3D reconstruction of multiple views simultaneously and is thus complex and inadequate.

**Figure 1:**
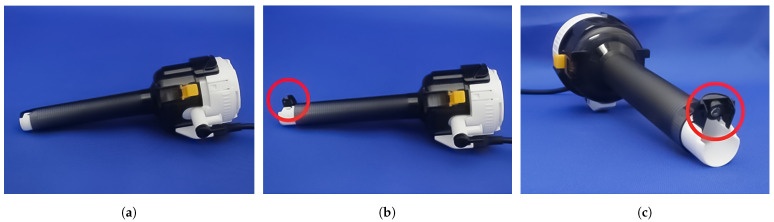
Trocar with camera (CARET): (**a**) retracted, (**b**) expanded and (**c**) frontal view.

In order to obtain an enhanced visual field, it is considered necessary to observe the abdominal cavity from new ports other than the laparoscope. Therefore, Okubo et al. [11] developed a trocar with a camera called CARET that can be used to observe the surgical site from different viewpoints. CARET has a retractable camera attached to its tip as shown in Figure 1. This camera can be retracted during trocar insertion and removal and expanded during the surgical time as shown in Figure 1c. CARET allows surgeons to acquire images while maintaining its original function. By using several CARET devices, it is possible to obtain intra-abdominal images from multiple viewpoints while maintaining the minimally invasive nature of laparoscopic surgery. Initial experiments confirmed that with the images obtained from a CARET, it was possible to observe parts that could not be observed from the laparoscope point of view, which enhances the accuracy and efficiency of operations such as suturing. However, it was pointed out that simply presenting a laparoscope and multiple viewpoint images on the monitor is not enough to expand the surgical field of view, and it is difficult to grasp the spatial relationship of the images.

Takada et al. [12] proposed a method for real-time surgical view expansion using multiple CARET devices. In this method, intra-camera tracking is used to estimate the relations between consequent camera frames. The correspondence between the different cameras is calculated at the initialization stage and is updated frequently when there is enough overlap between the views. This method can maintain the extended view even when there is no overlap between cameras. However, the error accumulation over a long time leads to inaccurate mosaicking results. In [13], an enhanced method was proposed by the same team to enhance the tracking accuracy and to construct a more accurate expanded view. In the method, image enhancement and feature point filtering are utilized to reduce the error. The evaluation results showed that this method performed better than the original one in most cases and can produce an expanded view at a rate of 20.4 fps. However, both methods assume that the environment is planar and they depend on 2D feature points tracking and homography transformation, which leads to inaccurate results. Additionally, the method can fail due to the difficulty of finding matches between the feature points at the initialization phase.

Therefore, in this work, we propose a field-of-view expansion approach for minimally invasive surgery based on 3D camera localization using Visual Simultaneous Localization and Mapping (vSLAM). We present a 2D dynamic expanded view instead of a dense 3D reconstruction. The proposed approach extends the ORB-SLAM [14] algorithm for multiple cameras’ pose estimation and tracking in the 3D space. A new algorithm is proposed to accurately calculate the relative camera positions and orientations and to combine different 3D maps.

This article is organized as follows: the proposed field-of-view expansion approach is presented in Section 2. The evaluation results using an ideal point cloud as well as a vSLAM point cloud, the evaluation of the proposed presentation approaches and the comparative results are presented in Section 3. In Section 4, the results using a human body model and in vivo data are discussed, and the article is finally concluded in Section 5.

## 2. The Proposed Field-of-View Expansion Approach

**Figure 2:**
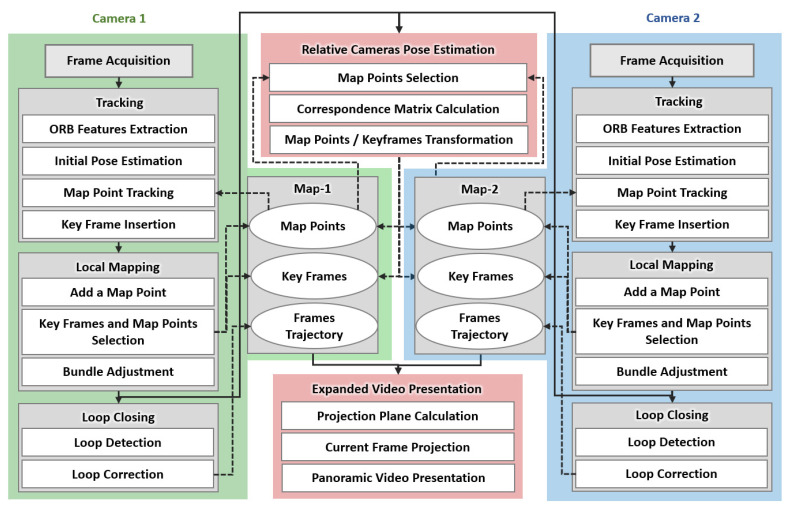
The pipeline of the proposed approach: three threads of Visual Simultaneous Localization and Mapping (vSLAM) for each camera, one thread for calculating the transformation between cameras and one for constructing the expanded view.

Since the conventional vSLAM is limited to the localization and mapping of a single monocular or stereo camera, we propose a vSLAM method for multi-camera setup where there is no constant baseline between cameras. Figure 2 shows the pipeline of the proposed approach. It extends the monocular Oriented features from an accelerated segment test and Rotated Binary robust independent elementary features—Simultaneous Localization And Mapping (ORB-SLAM) [14], which contains three parallel threads for tracking, mapping, and loop closing. In the proposed algorithm, the above three processes are performed for each camera, and a new thread is used to calculate the transformation from one camera to another. Another thread is also added to the proposed approach to produce the final expanded view, resulting in eight parallel threads. The details of the proposed approach will be presented in the following sub-sections.

### 2.1. Overview of the ORB-SLAM Algorithm

The ORB-SLAM map is composed of 3D points, key frames, and frame trajectories. The map points are 3D points in the world coordinate system, and each map point has a coordinate value of (x,y,z). The key frames are used to construct the 3D map points that are associated with the ORB [15] feature points in each key frame. ORB is an oriented multi-scale FAST [16] corner detector with an associated BRIEF [17] descriptor. A Bag of Words (BoW) [18] technique based on the local ORB features histogram is used to calculate the similarity between key frames. The frame trajectory is calculated from a graph with key frames as nodes.

In ORB-SLAM, the tracking is the main thread. It initializes the system, computes the pose of the first two key frames, and calculates 3D map points. In order to construct an initial 3D map, the relative pose between two frames is calculated. In the tracking thread, the pose of the camera is also estimated at each frame. This thread has four processes; ORB features detection, initial pose estimation, map point tracking, and key frame insertion. For point tracking, the adjacent key frames are selected by utilizing the roughly estimated initial camera pose and corresponding points are calculated. Afterwards, the initial camera pose is improved by robust nonlinear minimization of the Huber function [14].

The 3D map is updated each time a key frame is inserted. Specifically, map points are added, key frames are selected, and a bundle adjustment is performed. In the map point addition, triangulation is performed between adjacent key frames, and only corresponding points satisfying epipolar constraints are added. The bundle adjustment becomes complicated as the number of key frames increases. Therefore, the redundant key frames are detected and deleted. A key frame is considered redundant if 90% of the its map points exist in another three or more key frames. vSLAM accumulates errors sequentially and has the problem of mis-recognizing the same place at different time points. In order to solve this problem, loops are detected and corrected using loop closing for the latest key frame after local mapping. Candidate frames are obtained using BoW vector similarity evaluation, corresponding points with the latest key frame are searched, and a similarity transformation matrix is calculated. Afterwards, camera pose and map points are optimized by propagating the similarity transformation to the adjacent key frames.

### 2.2. Relative Pose Estimation Algorithm for Multi-Camera Setup

In order to extend the monocular ORB-SLAM [14] to a multi-camera setup, we propose a method for calculating the relative position and orientation between cameras. This method is based on Horn’s method [19] to estimate the transformation between point clouds. Horn proposed a 3D point cloud transformation method using quaternions [19]. If there are two point clouds $p_{i}$ and $q_{i}$ in different coordinate systems, the relationship between them can be estimated from three pairs of corresponding points. The center of gravity coordinates ${\overset{´}{p}}_{i}=p_{i}-\bar{p}$ and ${\overset{´}{q}}_{i}=q_{i}-\bar{q}$ can be used to estimate a unit quaternion $\dot{e}=\left(e^{0},e^{1},e^{2},e^{3}\right)$, where $\bar{p}$ and $\bar{q}$ are the centroid points of $p_{i}$ and $q_{i}$, respectively. Consequently, the rotation matrix, *R*, the scale, *s*, and the translation vector, *t*, of the transformation matrix can be calculated as described in [19].

In this section, we will first introduce methods for computing the transformation matrix using a set of automatically and manually selected points and then explain an improved method using quaternions filtering.

#### 2.2.1. Pose Estimation by Automatic Selection of Corresponding Points

To calculate the relative position and orientation between cameras, it is necessary to select the optimal key frame from the set of key frames of each camera. If the set of key frames of the first camera is $F_{i}^{1}\left(1≦i≦𝓃\right)$ and the set of key frames of the second camera is $F_{j}^{2}\left(1≦j≦𝓂\right)$, there are 𝓃×𝓂 key frame pairs. The ORB feature similarity is used to determine the corresponding map points in all pairs. If the number of corresponding points is greater than or equal to 20, which is empirically determined according to the average number of feature points in each frame, and the transformation matrix is calculated using Horn’s method. Three points are randomly selected from the corresponding points, and multiple attempts are made to obtain the transformation matrix. Afterwards, the re-projection error is calculated using each transformation matrix, and the number of inliers, i.e., points with a re-projection error below a certain threshold, is calculated. Then, we select the transformation matrix that yields the highest number of inliers as the final transformation matrix for this pair. If the obtained number of inliers is greater than or equal to 20, we consider this pair as optimal, and use its transformation matrix to calculate the relative position and orientation between cameras.

#### 2.2.2. Pose Estimation by Manual Selection of Corresponding Points

In the automatic selection method described above, the optimal pair of key frames is selected using the corresponding ORB points. However, if the angle of each camera with respect to the subject is significantly different, it is very difficult to automatically determine the corresponding points using ORB features. Therefore, we proposed a method to select the best corresponding points manually to solve this challenge.

A set of corresponding points on the key frames of each camera is manually selected by mouse click. Then, a feature point is identified around each selected point, and the corresponding map point is obtained. As described in Algorithm 1, three points are randomly selected from a set of points, and the transformation matrix including the rotation matrix, scale, and translation vector is calculated using Horn’s method. The re-projection error is calculated using this transformation matrix, and the corresponding points whose error is within the threshold are considered as inliers. Iterative calculation is performed, and the transformation matrix that maximizes the number of inliers is used as the final correspondence matrix.
**Algorithm 1:** Pose estimation by manual selection of corresponding points **Input**: a set of N corresponding points selected manually from two cameras.
 **Output**: the best transformation matrix which maximizes the number of inliers.
 Initialization:the best matrix =Ithe best no. of inliers =0
 Procedure:Find the nearest feature points to the corresponding points.Obtain the map points that represent the feature points determined in Step 1.Randomly select three points out of N points.Calculate the transformation matrix using Horn’s method.Calculate the no. of inliers based on the re-projection error.If no. of inliers > the best no. of inliers, set the best no. of inliers = no. of inliers and the best transformation matrix = the transformation matrix.If there is a group of points that have not been selected before, go to Step 3. Else, return the best transformation matrix.

#### 2.2.3. Improved Pose Estimation Method

In the previous pose estimation methods, only three points are randomly selected from a set of N points and this may affect the accuracy of the transformation matrix calculation due to outliers. In this section, therefore, we improve Horn’s method [19] by introducing a quaternion filtering algorithm. As described in Algorithm 2, ${}_{N}C_{3}$ quaternions ${\dot{e}}_{i}=\left(e_{i}^{0},e_{i}^{1},e_{i}^{2},e_{i}^{3}\right)$ are calculated using all possible three-point combinations. The median quaternion $\left(e_{m}^{0},e_{m}^{1},e_{m}^{2},e_{m}^{3}\right)$ is then calculated from the set of quaternions ${\dot{e}}_{i}$, and the range ${}^{𝓁}$ defined by (1) and (2) is used for outliers removal. The median is selected in this work as it helps to reject the outliers and concentrate on the correct points only.

(1) $$e_{m}^{𝓁}-\frac{\sigma^{𝓁}}{𝒹}\leq range^{𝓁}\leq e_{m}^{𝓁}+\frac{\sigma^{𝓁}}{𝒹},\;\left(𝓁=0,1,2,3\right)$$

(2) $$\sigma^{𝓁}=\sqrt{\frac{1}{n}\sum_{i=1}^{n}{\left(e_{i}^{𝓁}-\overset{-}{e^{𝓁}}\right)}^{2}},\;\left(𝓁=0,1,2,3\right)$$

where 𝒹 is a hyper-parameter to control the removal range. Increasing its value reduces the number of quaternions that are kept. $\bar{e^{𝓁}}$ is the mean value of each element, and *n* is the number of elements, in this case $n{=}_{N}C_{3}$.

After removing the outliers, we calculate a new representative quaternion as the mean of all quaternions within the range defined by (1). From this representative quaternion, the rotation matrix *R* is calculated as in [19]. We then use the rotation matrix *R* and the center of gravity coordinates $\overset{´}{p_{i}}$ and $\overset{´}{q_{i}}$ of all points to calculate the scale and the translation vector.
**Algorithm 2:** Improved pose estimation algorithm **Input**: a set of N corresponding points selected manually from two cameras.
 **Output**: improved transformation matrix based on all groups of points.
 Procedure:Find the nearest feature points to the corresponding points.Obtain the map points that represent the feature points determined in Step 1.Randomly select three points out of N points.Calculate quaternions.If there is a group of points that have not been selected before, go to Step 3. Else, continue.Calculate median of quaternions and set range using (1) and (2).Create new quaternion (mean within the range).Calculate the rotation matrix using the new quaternion calculated in Step 7.Calculate the centroid of the N points and relative coordinates, then calculate the scale and translation using Horn’s method [19].Calculate the final improved transformation matrix.

### 2.3. Displaying the Expanded View

To present an expanded view from different viewpoints, we propose an approach to create mosaicking images in real time. In the previous research, the ORB-SLAM [14] viewer can display the position and orientation of the current frame, the position and orientation of the key frames, and the map points. However, in this work, we extended the viewer by superimposing images from two cameras. Open Graphics Library (OpenGL) [20] is utilized to produce the final expanded view. We use texture mapping to draw camera images on GL space in real time. First, a memory area called a texture object is secured, and the camera image to be pasted is stored. On the other side, in the GL space, a polygon is defined for pasting the texture from camera image. By associating GL polygons and texture vertices, one frame can be rendered and by repeating this process, the camera image can be drawn in the GL space.

**Figure 3:**
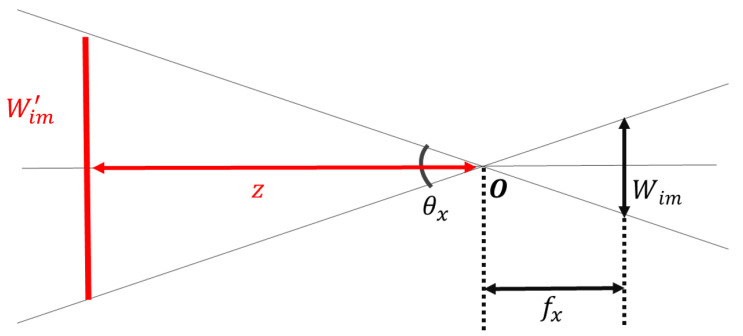
Pinhole camera model observed from the *y*-axis direction: $\theta_{x}$ is the angle of view in the *x*-axis direction, $f_{x}$ is the focal length along the *x*-axis, $W_{im}$ is the width of the image, and $W_{im}^{{}^{\prime }}$ is the width of the image on the projection plane.

In vSLAM, the camera’s self-position is defined as the camera’s principal point. Therefore, it is necessary to define the plane on which the camera image is projected and its size using camera parameters. Figure 3 shows the pinhole camera model observed from the *y*-axis direction. *O* is the principal point, $\theta_{x}$ is the angle of view in the *x*-axis direction, $f_{x}$ is the focal length along the *x*-axis, $W_{im}$ is the width of the image, and $W_{im}^{{}^{\prime }}$ is the width of the image on the projection plane. Here, if the distance *Z* from the principal point to the projection plane is used as a parameter, the width $W_{im}^{{}^{\prime }}$ of the projected image can be defined as in (3).

(3) $$W_{im}^{{}^{\prime }}=2Z\tan\frac{\theta_{x}}{2}=\frac{ZW_{im}}{f_{x}}$$

Similarly, if $\theta_{y}$ is the angle of view in the *y*-axis direction, $f_{y}$ is the focal length along the *y*-axis, and the height of the image is $H_{im}$, the height of the projected image, $H_{im}^{{}^{\prime }}$, can be calculated as in (4).

(4) $$H_{im}^{{}^{\prime }}=2Z\tan\frac{\theta_{y}}{2}=\frac{ZH_{im}}{f_{y}}$$

**Figure 4:**
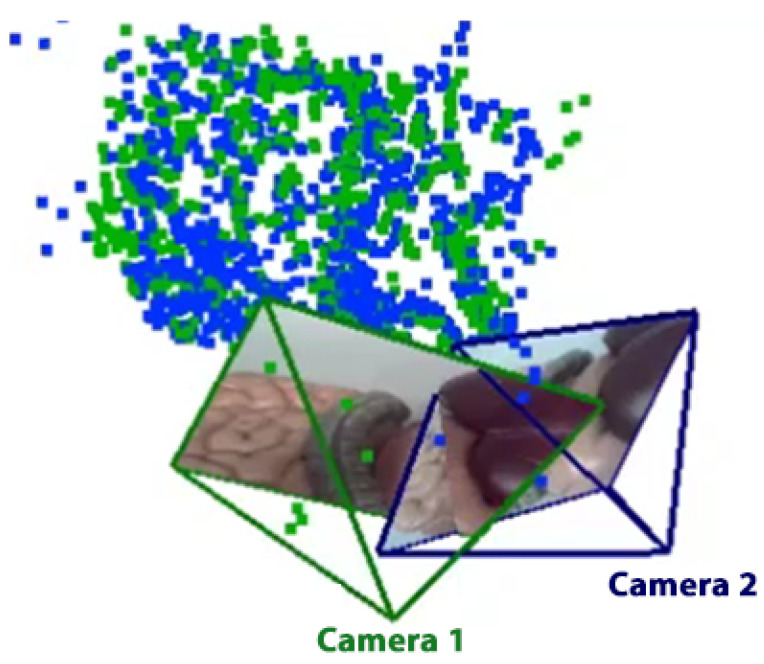
Multiple camera frames projection: visibility degradation due to overlap. The green point cloud is the map of camera 1 while the blue cloud is the transformed map of camera 2.

From Equations (3) and (4), the projection plane can be determined using the known camera parameters, which include the focal length and image size, and an empirical distance of the projection plane, *Z*. Accordingly, camera parameters of different CARET devices can be used to superimpose multiple video frames at the same time to construct a wider view, as shown in Figure 4. However, a clarity problem occurs when images from different cameras overlap. Therefore, we propose and examine different video presentation methods, planar projection, overlap removal and cylindrical projection, to increase the clarity in the case of views’ overlap.

#### 2.3.1. Planar Projection

**Figure 5:**
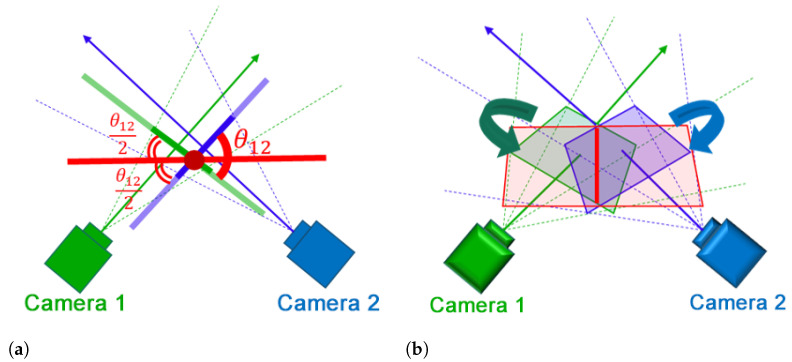
Constructing the expanded view using planar projection: (**a**) An abstract illustration of the intersection of planes and the angle between them, and (**b**) An illustration of the new projection plane shown in red.

Instead of using two different planes, one for each camera, we propose a method to create a single projection plane according to the intersection of camera planes. In Figure 5, camera 1 and camera 2 are represented using green and blue colors, respectively. The optical axis of each camera is represented using a solid straight arrow originating from the camera’s principle point. The four dotted lines connect the camera principle point to the vertices of the corresponding camera image. In Figure 5a, the solid lines perpendicular to camera’s optical axis represent its projection plane. The distance between the projection plane and the principal point is determined by the parameter *Z* used in Equations (3) and (4).

The intersection of the image plane $P_{1}$ of camera 1 and the image plane $P_{2}$ of camera 2 shown by the solid red line in Figure 5b is calculated. If $n_{1}$ is the normal vector of $P_{1}$, $n_{2}$ is the normal vector of $P_{2}$, and their distances from the origin to the planes are $h_{1}$ and $h_{2}$, respectively, the intersection line can be estimated by (5).

(5) $$l\left(\tau \right)=\frac{h_{1}-h_{2}\left(n_{1}\cdot n_{2}\right)}{1-{\left(n_{1}\cdot n_{2}\right)}^{2}}n_{1}+\frac{h_{2}-h_{1}\left(n_{1}\cdot n_{2}\right)}{1-{\left(n_{1}\cdot n_{2}\right)}^{2}}n_{2}+\tau \left(n_{1}\times n_{2}\right)$$

where τ is a parameter that determines the length of the line. The normal vectors $n_{1}$ and $n_{2}$ are used to estimate the angel between the two planes, $\theta_{12}$, as in (6).

(6) $$\theta_{12}=\arccos\left(\frac{n_{1}\cdot n_{2}}{\left|n_{1}\right|\left|n_{2}\right|}\right)$$

Accordingly, a new projection plane is created that includes the intersection line estimated in (5), and that has an angle $\theta_{12}/2$ with the projection plane of each camera. The camera image is projected on the new plane by calculating the intersection between this plane and the lines that connect the camera principle point to its image vertices, the dotted lines in Figure 5b. The intersection of the plane and each line is obtained using Equation (7).

(7) $$X_{in}=X_{0}+\frac{h-n\cdot X_{0}}{n\cdot m}m$$

where $X_{in}$ is the intersection point, n is the normal vector of the newly defined plane, *h* is the distance from the origin to the plane, $X_{0}$ is an arbitrary point on the straight line, and m is the direction vector of the line.

#### 2.3.2. Overlap Area Removal

**Figure 6:**
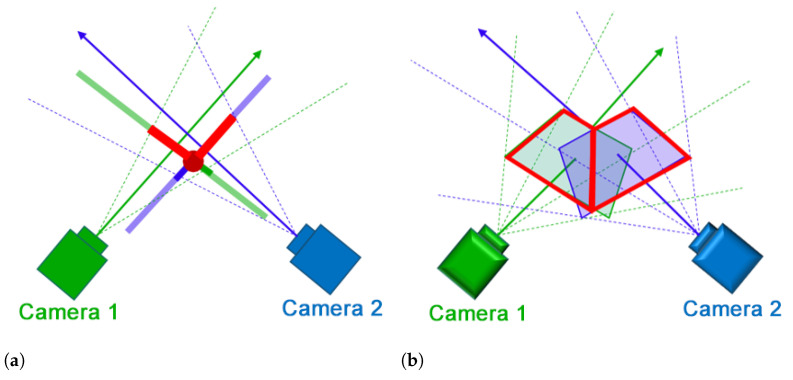
Constructing the expanded view using overlap area removal: (**a**) Abstract illustration of the intersection line as a red circle and the camera planes as thick red lines, and (**b**) shows the video display area after removing the overlap area as a red polygon.

**Figure 7:**
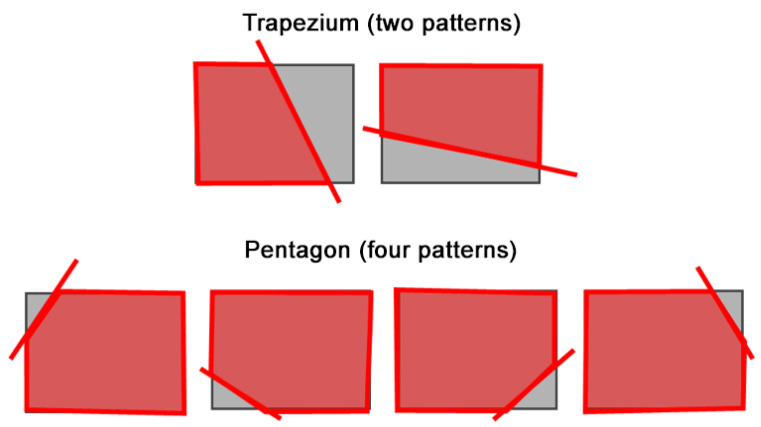
Different cases of camera frames after overlap area removal.

We propose a method to combine different views by removing the overlap area that appears in both views. To determine the overlap area, the intersection line is calculated using (5) and the overlap area is removed accordingly. The green and blue cameras shown in Figure 6 indicate camera 1 and camera 2, respectively. The solid arrow is the optical axis, the dotted lines are the straight lines connecting the principle camera point and the vertices of the camera image and the thick solid line represents the camera image plane. The intersection line between the camera image planes is represented by a red circle in Figure 6a and a red line in Figure 6b, and the camera image after cropping is represented by a red line. As shown in Figure 7, the shape of the camera image polygon after removing the area of overlap is one of six shapes: two trapezoids and four pentagons. We cut and transform texture objects according to their shape. By associating the deformed texture object with the polygon vertices of the camera image in the GL space, an image with the overlap region cut out is rendered.

#### 2.3.3. Cylindrical Projection

**Figure 8:**
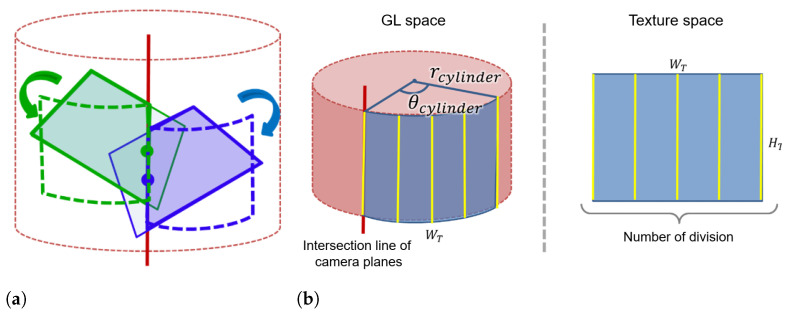
Constructing the expanded view using cylindrical projection: (**a**) is an overview of the projection method and (**b**) illustrates the approximation of the cylindrical surface by texture split.

Influenced by the fact that the abdominal area can be considered as a cylinder, we propose a method to project the extended view on a cylindrical surface. We define a cylinder that touches the projection plane used for the planar projection method. In Figure 8a, the red solid line is the intersection line between the two camera image planes defined by Equation (5), and the red dotted line is the cylinder that touches the plane. At the intersection line between camera image planes, the midpoint of the line segment from the upper side to the lower side of the camera image is calculated. The camera image is then projected onto the cylindrical surface based on the midpoint shown in Figure 8a. In GL space, OpenGL texture mapping needs a two-dimensional image. Accordingly, instead of using an exact cylinder, we define a polygonal cylinder with a rectangular side as an approximate cylinder. As shown in Figure 8b, each camera image is divided into rectangular strips that are projected one by one onto the approximate cylinder in the GL space. Increasing the number of strips results in a smooth cylindrical surface whereas it increases the computation time. To project an image of width $W_{T}$ that matches the arc length on the approximate cylinder, the approximate cylinder radius $r_{cylinder}$ is defined as in (8) with the angle $\theta_{cylinder}$ that determines the projection range.

(8) $$r_{cylinder}=W_{T}\frac{360}{2\pi \theta_{cylinder}}$$

### 2.4. Evaluation Metrics

Zero-mean Normalized Cross-Correlation (ZNCC) and Mutual Information (MI) were used for qualitative evaluation of the proposed video presentation methods. ZNCC was used to measure images’ similarity as in (9).

(9) $$ZNCC\left(I,J\right)=\frac{\sum_{i}\sum_{j}\left(I\left(i,j\right)-\bar{I}\right)\left(J\left(i,j\right)-\bar{J}\right)}{\sqrt{\sum_{i}\sum_{j}{\left(I\left(i,j\right)-\bar{I}\right)}^{2}\sum_{i}\sum_{j}{\left(J\left(i,j\right)-\bar{J}\right)}^{2}}}$$

where I(i,j) and J(i,j) are the pixel values at position (i,j) of the images I and J, respectively, and $\bar{I}$ and $\bar{J}$ are the average of all pixels. Since the cross-correlation used in ZNCC is calculated after normalizing each pixel using the average of all pixels, it is less affected by noise and the background of the entire image. ZNCC has a maximum value of 1 when the two images are identical, and it is zero when the two images are irrelevant.

Mutual information measurement is based on the concept of entropy and can be used to measure images’ similarity. The mutual information is maximal when the two images are the same, and it is zero when the two images are irrelevant. If we consider image pixels as the events $I_{𝒾}$ and $J_{𝒿}$ for images I and J, respectively, the MI is calculated as in (10).

(10) $$\mathrm{MI}\left(I,J\right)=\sum_{𝒾}^{255}\sum_{𝒿}^{255}P\left(I_{𝒾},J_{𝒿}\right)\log_{2}\frac{P(I_{𝒾},J_{𝒿})}{P\left(I_{𝒾}\right)P\left(J_{𝒿}\right)}$$

where the joint appearance probability $P(I_{𝒾},J_{𝒿})$ is calculated by (11) and the probabilities $P(I_{𝒾})$ and $P(J_{𝒿})$ can be calculated by Equations (12) and (13), respectively.

(11) $$P\left(I_{𝒾},J_{𝒿}\right)=\frac{H(I_{𝒾},J_{𝒿})}{\sum_{𝒾}^{255}\sum_{𝒿}^{255}H\left(I_{𝒾},J_{𝒿}\right)}$$

where $H(I_{𝒾},J_{𝒿})$ is a two-dimensional joint histogram counting the frequency of pixel value combinations $\left(I_{𝒾},J_{𝒿}\right)$.

(12) $$P\left(I_{𝒾}\right)=\sum_{𝒿}^{255}P\left(I_{𝒾},J_{𝒿}\right)$$

(13) $$P\left(J_{𝒿}\right)=\sum_{𝒾}^{255}P\left(I_{𝒾},J_{𝒿}\right)$$

## 3. Results

In this section, the evaluation results of the proposed view expansion approach are presented. All experiments were conducted using OpenCV 3.2 and Visual C++ 2015 on a Core I7 (6700, 3.4 GHz) machine with 16 GB RAM and a GeForce GTX 970 GPU.

### 3.1. Evaluation of the Pose Estimation Using Ideal Point Cloud

In this section, the evaluation results of the pose estimation methods using an ideal point cloud are presented. Because the map points obtained by ORB-SLAM do not have the scale information, a random point cloud of 10 points was created and used to evaluate the transformation matrix calculation methods. We applied the following procedure to construct the ideal and transformed point clouds and evaluate the accuracy of the pose estimation methods:

- Randomly create an ideal point cloud to represent camera 1 (range x∈[−5,5], y∈[−5,5], z∈[−5,5]).
- Randomly select rotation angles (roll, pitch and yaw) within the range [−π,π], a scale *s* within the range [0,5] and a translation *t* within the range [−5,5].
- Calculate the rotation matrix *R* from the roll, pitch and yaw angles.
- Transform the ideal point cloud created in (a) using *R*, *s* and *t*.
- Add noise assuming observation error to the transformed point cloud (range [−0.1s,0.1s])
- Estimate the relative transformation matrix between the ideal point cloud, which represents camera 1, and the transformed point cloud, which represents camera 2.
- Transform the point cloud obtained from step (e) back using the estimated matrix.
- Compare the estimated transformation matrix to the original one and evaluate the re-projection error.

After estimating the rotation matrix, the scale and the translation vectors, they were compared to the original values and the estimation error was calculated. The proposed estimation method using Horn’s approach and the proposed improved approach were compared as shown in Table 1 and Table 2 for two different sets of points. The parameters for removing outliers in (1) were empirically selected as 𝒹=2 and 𝒹=4. The original values are presented in the first row, the estimated values are presented in the second, fourth and sixth rows and the estimation error for each parameter is shown in the third, fifth and seventh rows. The first, second and third columns show the results for the components of the rotation vectors $\left(\omega_{x},\omega_{y},\omega_{z}\right)$ calculated from the original rotation matrix *R* and the estimated one. The rotation angle is presented in the fourth column and the angle between the rotation vectors, $\omega ,\overset{´}{\omega }$, which was calculated using (14) is shown in the fifth column. The sixth and ninth columns show the scale *s* and the components of the translation vector *t*, respectively. For each item, the method that had the smallest error is shown in bold.

(14) $$a=\cos^{-1}\left(\frac{\omega \cdot \overset{´}{\omega }}{\left|\omega \right|\left|\overset{´}{\omega }\right|}\right)$$

**Figure 9:**
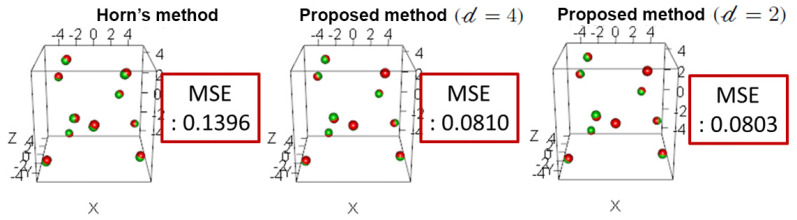
Comparison of relative pose estimation methods using a set of ideal points.

From this result, it is confirmed that the proposed enhanced estimation method has a smaller estimation error and a higher accuracy than Horn’s method. In Figure 9, the red points are the point cloud that represents camera 1, and the green points are the point cloud that represents camera 2 after transformation using the estimated correspondence matrix. The mean square error (MSE) between point clouds was calculated for each method. The results indicate that the MSE of the proposed improved pose estimation method is significantly lower than the MSE of Horn’s method.

### 3.2. Evaluation of the Pose Estimation Using vSLAM Map Point Cloud

The vSLAM map points that correspond to camera 1 and camera 2 were obtained, and the relative transformation matrix was then calculated using Horn’s method and the proposed improved method. Because it is difficult to obtain the ground truth of the transformation matrix, the evaluation was performed using the mean square error between the transformed point clouds and their variance. In the improved method, the outlier removal parameter was set to 𝒹=2. The outlier removal result and the transformation result using the estimated matrix are presented below.

**Figure 10:**
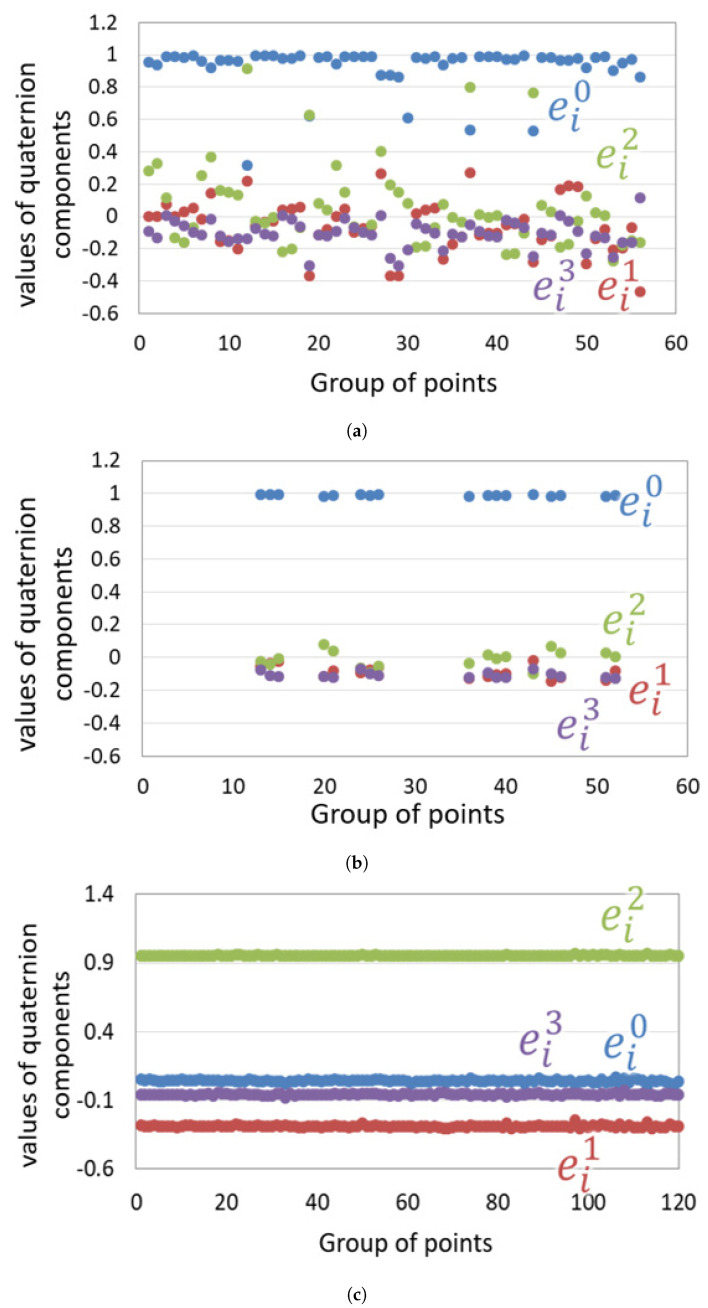
Quaternions calculated using different groups of corresponding vSLAM map points (**a**) without outlier removal, (**b**) after outlier removal and (**c**) ideal set of points.

**Figure 11:**
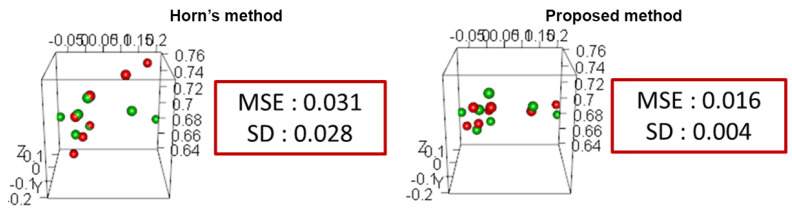
Comparison of the relative pose estimation methods using a set of vSLAM map points.

To investigate the effect of outlier removal using the proposed improved pose estimation method, ${}_{N}C_{3}$ quaternions ${\dot{e}}_{i}=\left(e_{i}^{0},e_{i}^{1},e_{i}^{2},e_{i}^{3}\right)$ were calculated from N pairs of corresponding vSLAM map points as shown in Figure 10a. Table 3 shows the mean, median, standard deviation, maximum and minimum values of each component of the quaternion. For comparison, we calculated the quaternion using the ideal point cloud used in the previous evaluation experiment as shown in Figure 10c and Table 3. Unlike the ideal point cloud, the quaternions calculated from the vSLAM map points had a large variation for all elements. Figure 10b shows the estimated quaternions after the outlier removal using (1). These results indicate that the improved method is more robust and can estimate the relative transformation matrix more accurately. Figure 11 shows the results of vSLAM map points transformation using the estimated matrix. Horn’s method and the proposed method were compared. The red dots are the point cloud that represents camera 1, and the green dots are the point cloud that represents camera 2 after transformation using the estimated transformation. MSE is the mean square error between point clouds in each method, and SD is their variance. As can be noticed, the MSE of the proposed improved method is about half that of Horn’s method, and the variance was significantly reduced.

### 3.3. Evaluation of the Proposed Display Methods

**Figure 12:**
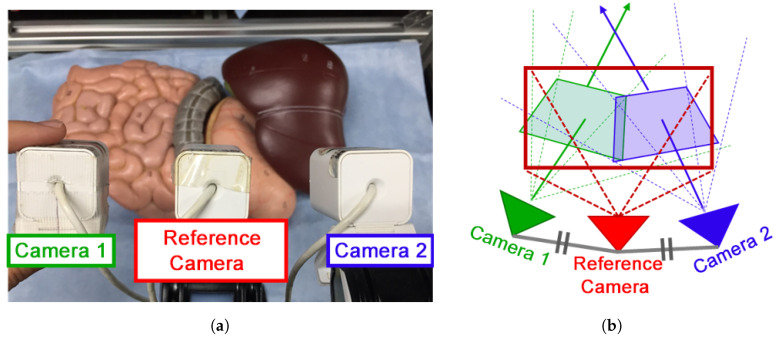
Experimental setup for presentation methods evaluation: (**a**) experiment environment (human model); (**b**) cameras’ setup.

In this section, the quantitative and qualitative evaluation of the proposed video presentation methods will be presented. For the qualitative evaluation of the proposed presentation methods, we designed the experiment shown in Figure 12a. Three USB cameras, ELECOM’s UCAM-DLF30, with a resolution of 640×480 and a frame rate of 30 fps, were used; two cameras were installed to the left and the right to simulate the CARET devices and one at the middle to capture a reference image. The reference camera was placed in the middle point between the other cameras as shown in Figure 12b. All cameras focused on the liver, stomach, and upper small and large intestines of the human model. Three expanded views were obtained using the proposed presentation methods and the overlap with the reference image was selected manually in each case. Finally, image similarity was evaluated using ZNCC and MI metrics. Table 4 shows the ZNCC and MI scores for each presentation method. The results of this experiment indicate that the overlap removal presentation method obtained the best ZNCC and MI scores.

**Figure 13:**
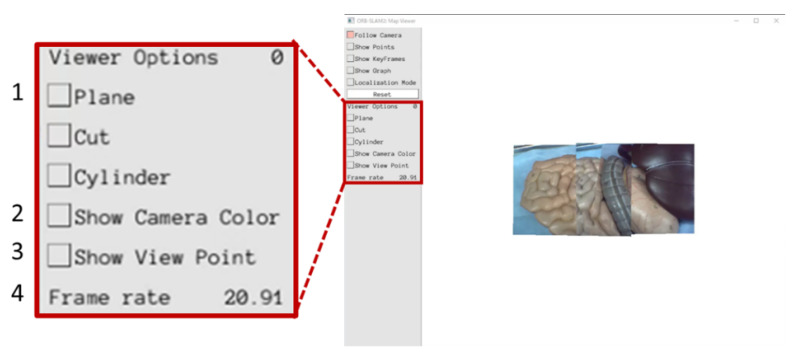
User interface for expanded view presentation.

We also evaluated the proposed presentation methods based on a specialist’s opinions. The user interface shown in Figure 13 was created in order to continuously construct the expanded view using the proposed video presentation methods. In Figure 13, “1” can be used to switch the video presentation method. By using “2”, the operator can display the border of the camera images in two different colors (camera 1: green and camera 2: blue). The number “3” indicates the viewpoint position and “4” indicates the frame rate. A Gastroenterologist observed the expanded views construed using the proposed presentation methods and commented on them. According to the specialist’s opinion, it was found that the stability of the resulting video is very important. From the stability point of view, both planar and cylindrical projection methods have many flickers, suggesting that the images are less visible to the operator. The overlap removal method has less flicker than the other two methods, and a stable video output is possible, which makes it more suitable in real situations.

### 3.4. Comparative Results

**Figure 14:**
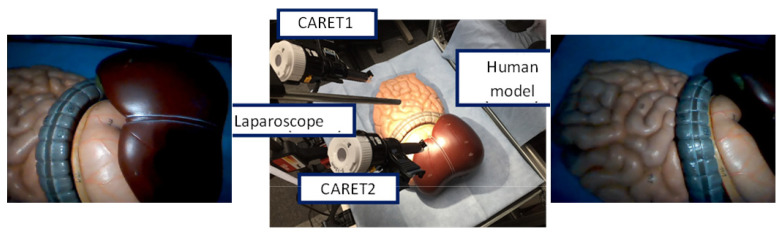
Human model experiment setup: human model environment with two CARET devices and a laparoscope as a light source in the middle; CARET-1 image on the left side and CARET-2 image on the right side.

In this section, we compare the proposed field of view expansion approach to the improved hybrid tracking and matching algorithm [13] and the traditional mosaicking approach using a human model as well as in vivo data. Figure 14 shows the setup and sample frames for the human model experiment. The resolution of the CARET was 640 × 480 and the frame rate was 30 fps. A laparoscope was used as a light source only.

**Figure 15:**
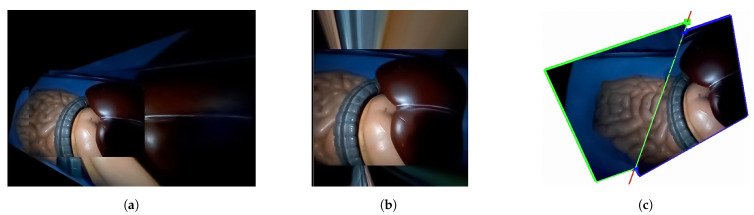
Results of the human model experiment: (**a**) the results of the conventional approach, (**b**) the results of the hybrid tracking and mosaicking approach and (**c**) the results of the proposed approach.

Figure 15 shows the results of the human model experiments. As can be noticed from Figure 15a, the mosaicking result of the traditional approach was not accurate due to the small number of feature points and the difficulty of the matching process. Figure 15b also shows that it is difficult to produce the extended view using the hybrid tracking and mosaicking approach. Figure 15c shows the results of the proposed approach. These results indicate that the proposed approach is the only one that can produce a correct expanded view.

**Figure 16:**
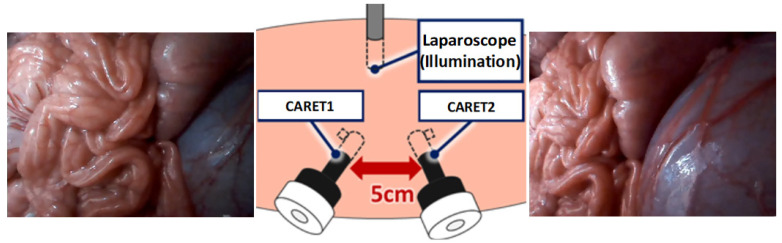
In vivo experimental setup with two CARET devices and a laparoscope as a light source; CARET-1 image on the left side and CARET-2 image on the right side.

**Figure 17:**
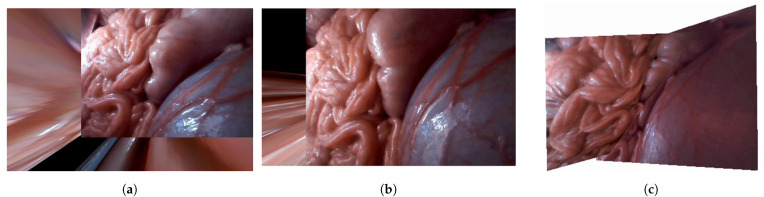
Results of in vivo experiment: (**a**) the results of the conventional approach, (**b**) the results of the hybrid tracking and mosaicking approach and (**c**) the results of the proposed approach.

The setup and sample frames for the in vivo experiment are shown in Figure 16. The results of the in vivo experiments shown in Figure 17 confirm the same conclusions we obtained from previous results using the human body model. It is difficult to construct the expanded view using either the traditional mosaicking technique or the improved hybrid tracking and mosaicking algorithm as shown in Figure 17a,b. We were only able to produce the expanded view properly by using the proposed approach as shown in Figure 17c.

## 4. Discussion

In Section 3.1 and Section 3.2, the relative position and orientation estimation methods were evaluated. The evaluation results indicated that the proposed method has a smaller mean square error and standard deviation than Horn’s method and it can efficiently remove the outliers. It was also proven that the transformation matrix calculation considering the entire point cloud is possible. This method has more iterations than Horn’s method and this may cause an increase in computation time. However, the inter-camera transformation matrix calculation is performed only once during vSLAM processing, and since it is calculated in parallel processing for the other threads, accuracy should be given priority over calculation time.

In Section 3.3, we presented a quantitative evaluation of the video presentation methods. In planar and cylindrical projection methods, the image is projected on a new plane or curved surface, which produces an unnaturally deformed image, and the view angle of the image is reduced. In addition, the planar projection is inaccurate when the angle between the two cameras increases because the new plane cannot accurately represent both image planes. On the other hand, the overlap region removal method uses a plane calculated from the camera’s own position without any deformation, and therefore it has a higher degree of matching than the other two methods, suggesting that a more natural expanded view can be achieved. The qualitative evaluation of the video presentation method indicated that the stability of the image is very important and the overlap area removal is the most suitable presentation method.

Furthermore, comparative evaluation experiments were performed to compare the proposed approach to the conventional mosaicking approach and the improved hybrid tracking and mosaicking approach [13]. In experiments using CARET devices, the two other methods could not output an expanded view. However, the proposed approach could always output it based on 3D map points. All results indicate the efficiency of the proposed approach and its ability to provide an expanded surgical view.

Tracking is not used in the traditional mosaicking approaches. The improved hybrid tracking and matching algorithm utilizes feature points tracking in 2D images, and the proposed approach is based on the tracking of the 3D map points. The traditional mosaicking approach extracts and matches the feature points at each frame, whereas the improved hybrid tracking and mosaicking approach performs these operations at the initial and update frames only. On the other hand, the proposed approach performs point clouds alignment only once based on the relative position and orientation between cameras. Enough image overlap at each frame is necessary for the traditional mosaicking approach, and it fails when there are not enough common features. The tracking approach requires enough overlap for initialization and update processes only, and can work even if there is less or no overlap. However, it depends on a flat surface in the calculation of the transformation and errors accumulate from one frame to another, making the update process essential. On the contrary, the proposed approach does not rely on overlapping images and considers the 3D nature of the environment. Therefore, it can produce more accurate results. The proposed approach could also work in real time at 21 fps and the hybrid mosaicking approach could work at 22 fps, whereas the traditional approach operates much slower at 10 fps.

In all experiments, we used two identical CARET devices, but in the future, we intend to consider the situation of using a combination of different cameras such as a CARET and a laparoscope, assuming an actual surgical environment. Moreover, we used the default parameters of ORB-SLAM, which were adapted in previous research. Compared with indoor or outdoor images where self-localization is generally performed, intra-abdominal images have extremely few feature points, and it is thought that changes to the imaging environment due to body movements and organ deformations are large. Therefore, it is necessary to adjust the parameters considering the characteristics of the intra-abdominal image.

## 5. Conclusions

Field-of-view expansion is very important for laparoscopic surgery. It can help to deliver safer surgeries and reduce the burden on surgeons. Therefore, we proposed a new field-of-view expansion approach using 3D map points obtained from vSLAM, which is a 3D self-position estimation method of the camera, and its tracking. Unlike the conventional vSLAM methods, which use a single monocular or stereo camera, we extended the vSLAM to use multiple cameras where the baseline between them is not constant. To extend the ORB-SLAM algorithm for a multi-camera setup, we proposed an algorithm to estimate inter-camera relative position and orientation. This algorithm estimates the optimal inter-camera transformation matrix by determining the optimal set of quaternions based on the corresponding 3D map points of the two cameras. Quantitative and qualitative evaluation of three different presentation methods indicated that the overlap area removal is the most suitable presentation method. Furthermore, comparative results of experiments using CARET devices indicate that unlike the compared methods, the proposed approach could always construct the expanded view using 3D map points. All results indicate the efficiency of the proposed approach and its ability to produce an expanded view in real time. In the future, we aim to investigate other video presentation methods to provide a better and a more stable visualization of the expanded view. 

## Figures and Tables

**Table 1:** Comparison of the relative pose estimation methods—first set of points.

	$\omega_{x}$	$\omega_{y}$	$\omega_{z}$	$\theta {[}^{\circ }]$	$a{[}^{\circ }]$	***s***	$t_{x}$	$t_{y}$	$t_{z}$
Ground truth	1.1644	1.7179	2.3026	177.605	−	0.5	4	5	−2
Horn’s method	1.1585	1.7389	2.2862	177.453	0.501631	0.4979	4.0061	4.9494	−2.0102
Error	−0.0059	0.021	−0.0164	−0.152		−0.0021	0.0061	−0.0506	−0.0102
Proposed method (𝒹=4)	1.1575	1.7313	2.2979	177.688	0.29074	0.4985	4.0647	5.0015	−1.9969
Error	−0.0069	0.0134	−0.0047	0.083		−0.0015	−0.0647	−0.0015	0.0031
Proposed method (𝒹=2)	1.1571	1.7264	2.3034	177.751	0.200769	0.4985	4.0011	4.9975	−1.9811
Error	−0.0073	0.0085	0.0008	0.146		−0.0015	0.0011	−0.0025	0.0189

**Table 2:** Comparison of the relative pose estimation methods—second set of points.

	$\omega_{x}$	$\omega_{y}$	$\omega_{z}$	$\theta {[}^{\circ }]$	$a{[}^{\circ }]$	***s***	$t_{x}$	$t_{y}$	$t_{z}$
Ground truth	−2.1374	−1.4560	1.0740	160.446	−	1.5	−3	−2	−2
Horn’s method	−2.1505	−1.4359	1.0556	160.029	0.60166	1.5077	−3.1509	−2.0992	−1.9921
Error	−0.0131	0.0201	−0.0184	−0.417		0.0051	−0.1509	−0.0992	0.0079
Proposed method (𝒹=4)	−2.1475	−1.4559	1.0578	160.533	0.38816	1.4971	−3.0295	−1.9922	−1.9528
Error	−0.0101	0.0001	−0.0162	0.087		−0.0019	−0.0295	0.0078	0.0472
Proposed method (𝒹=2)	−2.1531	−1.4479	1.0615	160.621	0.43790	1.4971	−3.0436	−2.0111	−1.9419
Error	−0.0157	0.0081	−0.0125	0.175		−0.0019	−0.0436	−0.0111	0.0581

**Table 3:** Comparison of the relative pose estimation methods.

	Quaternions Calculated from a Set of vSLAM Map Points.				Quaternions Calculated from a Set of Ideal Points.			
	$e^{0}$	$e^{1}$	$e^{2}$	$e^{3}$	$e^{0}$	$e^{1}$	$e^{2}$	$e^{3}$
Gold Standard	−	−	−	−	0.045	−0.289	0.954	−0.059
Mean	0.925	−0.072	0.059	−0.104	0.041	−0.288	0.954	−0.057
Median	0.978	−0.056	−0.001	−0.104	0.042	−0.289	0.954	−0.057
Std. dev.	0.134	0.181	0.256	0.079	0.008	0.007	0.002	0.006
Maximum	0.995	0.271	0.912	0.115	0.064	−0.246	0.967	−0.031
Minimum	0.318	−0.763	−0.277	−0.307	0.002	−0.302	0.950	−0.078

**Table 4:** Evaluation results of the proposed presentation methods. ZNCC: Zero-mean Normalized Cross-Correlation; MI: Mutual Information.

Method	Planar Projection	Overlap Area Removal	Cylindrical Projection
ZNCC	0.593	0.778	0.680
MI	1.065	1.262	1.141

## Data Availability

The data presented in this study are available on request from the corresponding author. The data are not publicly available due to ethical issues related to animal experiments and institutional rules.
